# A Cross‐Sectional Study on Gastric Diseases and FGIDs in Bangladesh: Insights Into Causes, Symptoms, and Medications

**DOI:** 10.1002/hsr2.71188

**Published:** 2025-08-19

**Authors:** Md. Shoriful Islam, Rashni Agarwala, Sozoni Khatun, Tonima Enam, Ayesha Siddika, Tasfia Saffat, Mst. Israt Jahan, Afia Ibnath Shimki, Azifa Akter, Mahajabin Snigdha, Rubaia Tasmin

**Affiliations:** ^1^ Department of Pharmacy Islamic University Kushtia Bangladesh

**Keywords:** capsule, FGIDs, gastritis, GERD, H_2_ blockers, obesity, PPIs, regurgitation, self‐medication

## Abstract

**Background and Aims:**

Acute gastritis affects roughly 8 out of every 1000 people, while chronic gastritis affects about 2 out of every 10,000 people worldwide. When something destroys or affects the mucosa lining the stomach, which protects it from the powerful stomach acid that breaks down food, the mucosa becomes inflamed, causing gastritis. A questionnaire‐based survey was designed to assess the epidemiology of gastritis based on the patient's symptomatology and potential causes of these symptoms, severity, current epidemiology, treatment, and assessment of the health‐related quality of life with response to treatment and medication awareness of gastritis‐related diseases in Bangladesh.

**Methods:**

A self‐reported, cross‐sectional study of 1051 gastric patients (57% male and 43% female) was conducted in Bangladesh using a simple random selection method with descriptive demographic and clinical characteristic statistics. Based on the availability of high‐quality data, we worked on several governmental and nongovernmental hospitals and clinics in eight randomly selected districts around the country.

**Results:**

With an 87.58% response rate (603 men and 448 females), 81% of participants were actual gastric patients, and the remaining 19% took gastric medicines with other medications for various diseases. Patients between the ages of 31 and 60 were the most likely to suffer from gastritis, where males are significantly more represented than females. A sedentary lifestyle (45%, *n* = 478) and obesity (28%, *n* = 294) are the most common causes, while regurgitation (64%, *n* = 677) and stomach bloating (58%, *n* = 609) are the most common symptoms. Surprisingly, 51% of patients were taking self‐medication, mostly (79.1%, *n* = 831) proton pump inhibitors (PPIs), a class of medications that come in solid dosage forms (capsule 63% and tablet 33%).

**Conclusion:**

This survey revealed the prevalence of gastritis and functional gastrointestinal disorders (FGIDs) in Bangladesh for the first time. The insight of this study will contribute to a better understanding of the current causes, epidemiology, diagnosis, symptoms, and treatment of gastritis in Bangladesh.

## 1 Introduction

The research on gastritis prevalence, a crucial study area, has revealed significant variations by demography and time. In 1968, Finnish rural research discovered a lower frequency of 53%, which increased with age [1]. A global analysis from 1990 to 2019 found an age‐standardized prevalence rate of 518.11 per 100,000 in 2019, with the highest rates among those aged 50–69 and in low sociodemographic index (SDI) regions [2]. These findings underscore the persistent burden of gastritis, particularly in specific age groups and areas, and highlight the need for targeted therapies and further research into prevalence patterns and risk factors.

Gastritis is the inflammation and swelling of the gastric mucosa [3], stomach lining inflammation that could be acute or chronic, especially in the upper abdomen, particularly in the epigastrium. Chronic gastritis is the most common form of gastritis, a chronic inflammatory condition of the gastric mucosa caused by *Helicobacter pylori* [4, 5]. Over 3 billion people are known to be infected by *H. pylori*, and most of these infections occur in developing countries [6]. Other common factors that can cause gastritis include autoimmune gastritis, taking nonsteroidal anti‐inflammatory drugs (NSAIDs), bile reflux, smoking, drinking alcohol, not drinking enough water, overeating dairy, leading an inactive lifestyle, getting older, eating an unbalanced diet, experiencing major surgery or mental or physical stress [4, 7].

The signs and symptoms of gastritis vary among individuals, and some may not have any symptoms. Functional gastrointestinal disorders (FGIDs) or gut–brain Interaction, updated through the Rome IV conference, scientifically categorized the (sub) types of disorders based on a biopsychosocial model [8]. Various symptoms originating in the gastrointestinal (GI) tract include abdominal pain, gastroesophageal reflux disease (GERD), diarrhea, constipation, loss of appetite, a complete feeling of stomach after a meal, heartburn, acid taste, chest pain, chronic cough, bloating, nausea, and vomiting [9]. These symptoms are manifested in a wide range of organic pathologies, including GI cancer, inflammatory bowel disease, celiac disease, peptic ulcer disease, and movement disorders such as gastroparesis [8].

Untreated gastritis may lead to peptic ulcer, chronic atrophic gastritis, gastric cancer, gastric bleeding, and vitamin C, vitamin D, vitamin B_12,_ folic acid, zinc, magnesium, and calcium deficiency [7, 10]. Treatment approaches differ from lifestyle and dietary changes to medication (antibiotics, proton pump inhibitors (PPIs), H_2_ blockers, antacids, vitamin supplementation, and prostaglandins) to immunomodulatory therapy and endoscopic therapy to surgery [7, 11, 12].

Oral formulations, absorption, distribution, metabolism, excretion (ADME) properties, formulation type, and drug design can significantly affect a drug's pharmacokinetics (PK). However, the use of oral medications must also consider that various factors such as age, sex, ethnicity, disease, and diet can alter the gastrointestinal (GI) environment, including peristalsis pattern, transit time, liquid volume, composition, pH, enzyme composition, and activity of the internal components, which may affect oral drug absorption [12].

The proportion of the population with FGIDs diagnosed by Rome IV and their cumulative impact on health impairment in Bangladesh remains unknown. Based on the interviewed patients' opinions, this study aimed to survey the causes, symptoms, and primary medicines used in gastritis and FGIDs prevalence.

## 2 Materials and Methods

### 2.1 Participants Clusters

We collected data based on patient‐reported symptoms, according to the Rome Foundation's committee of gastroenterologists and related academics in GI health. It is also a cost‐effective process. Common functional symptoms data were collected from the inpatients and outpatients who may take gastritis‐related drugs because of gastritis or any other medication for treating illness. 1200 randomly selected gastric patients (661 males and 539 females) were approached, and 1051 gastric patients were recruited to participate in the study in Bangladesh. The demographic characteristics are shown in Table 1 (Table S1).

**Table 1:** Demographic information of total participants with the number and percentages of males and females in each district.

District	Total no. (1051)	Male (603)	Female (448)
Kushtia	200	105 (53%)	95 (47%)
Jhenaidah	111	62 (56%)	49 (44%)
Jessore	131	60 (53%)	71 (47%)
Rajshahi	115	41 (36%)	74 (64%)
Bogura	120	104 (87%)	16 (13%)
Rajbari	110	73 (66%)	37 (34%)
Dinajpur	170	98 (58%)	72 (42%)
Dhaka	94	60 (64%)	34 (36%)

### 2.2 Data Collection and Survey Duration

A team of fourth‐year undergraduate students from the Department of Pharmacy at the Islamic University in Bangladesh conducted a personal interview. The written standard questionnaire was pre‐structured and comprised of dichotomous, multiple‐choice, and short questions about the causes, symptoms, and treatment of gastritis. The general inquiries include the patient's name, age, gender, weight, lifestyle, and knowledge of drugs and healthcare personnel. A paper‐pencil‐based questionnaire method was used to gather and record data. Gastritis status was determined by whether patients experienced signs and symptoms of gastritis that lasted less than a month, more than a month, or none at all, but were taking gastric medication. Since it is a cross‐sectional study based on symptomatic data, no follow‐up diagnostic and other test results exist. From February to April 2022, the survey was conducted via an offline, face‐to‐face, closed‐ended questionnaire form (Images S1 and S2).

### 2.3 Ethical Consideration

After explaining the aim and purpose of the study, patients gave their written consent and permission for the study. We consulted with the patients and their relatives who took care of them. We did not use emotionally loaded or biased words or phrases. There was no risk of physical harm to the participants, and no sensitive questions were asked during the interview. All information was standardized, and privacy was maintained under the guidelines of the “Islamic University Ethical Review Committee.” The ethics approval number for this study was FBS/EC/2020/03.

### 2.4 Exclusion and Inclusion

This study is restricted to patients with gastritis or taking medication for acid neutralization. During the sampling process, other patients were excluded.

### 2.5 Statistical Analysis

We performed an unpaired *t*‐test to check the statistical significance. Statistical analysis was performed using Microsoft Excel 2013 and GraphPad Prism 8 software.

## 3 Results

Gastritis is a common disease that affects people worldwide. It is associated with many troublesome symptoms, which can significantly impact health.

### 3.1 Age of Patients

**Figure 1:**
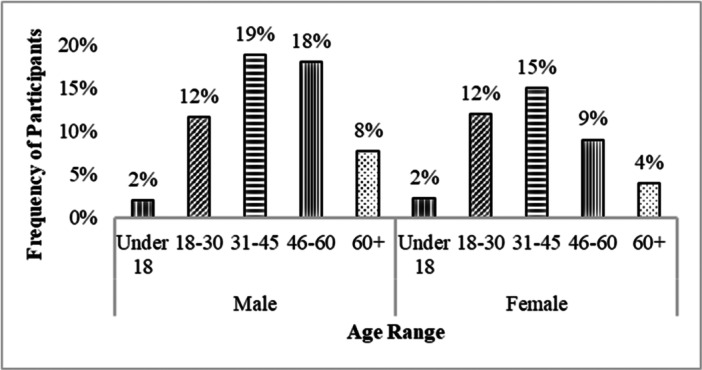
Percentage of gastric patients according to age ranges and sex. The five age ranges were from 8 months to 85 years old.

Most of the patients (81%) had gastritis, and the remaining 19% of patients used gastric medicines with other medications used for various diseases such as arthritis, asthma, diabetes, hypertension, and so on. Symptoms of gastritis are more common in adults aged 31–45. Those under the age of 18 (2%) were less likely to have gastritis symptoms than those over the age of 30 (30%). Similarly, males are significantly (*p* < 0.001) more represented than females in this 40‐ to 60‐year age group in the study population (Figure 1).

### 3.2 Common Causes of Gastritis

**Figure 2:**
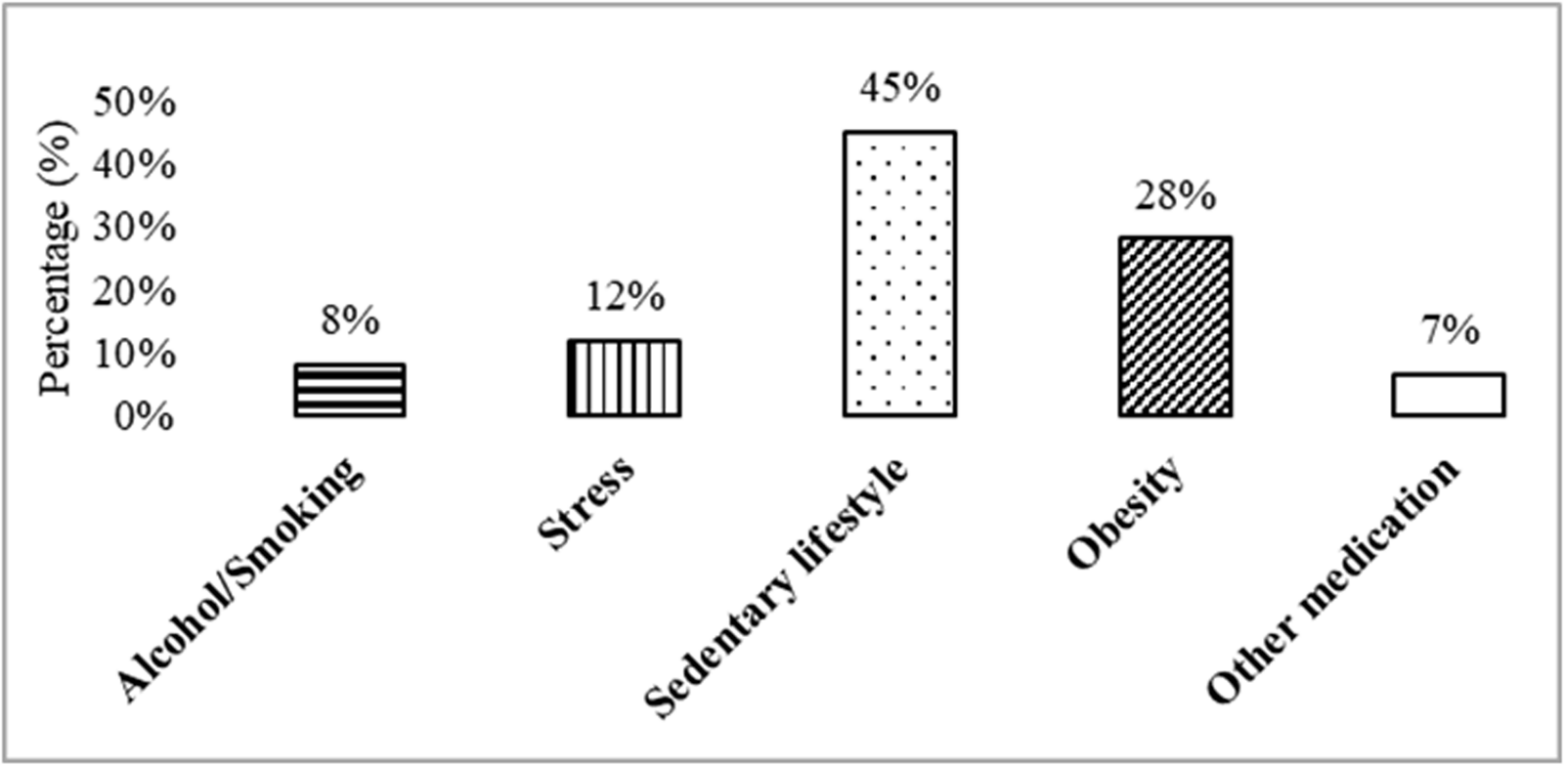
Common causes of gastritis reported by the participants (*n* = 1051).

The majority of men (66%) and all female (100%) participants said they did not drink alcohol or smoke cigarettes. Leading sedentary lifestyles (45%) is the most common cause of gastritis, followed by obesity (28%), stress (12%), alcohol/smoking (8%), and others (7%) (Figure 2).

### 3.3 Symptomatic Comparison

**Figure 3:**
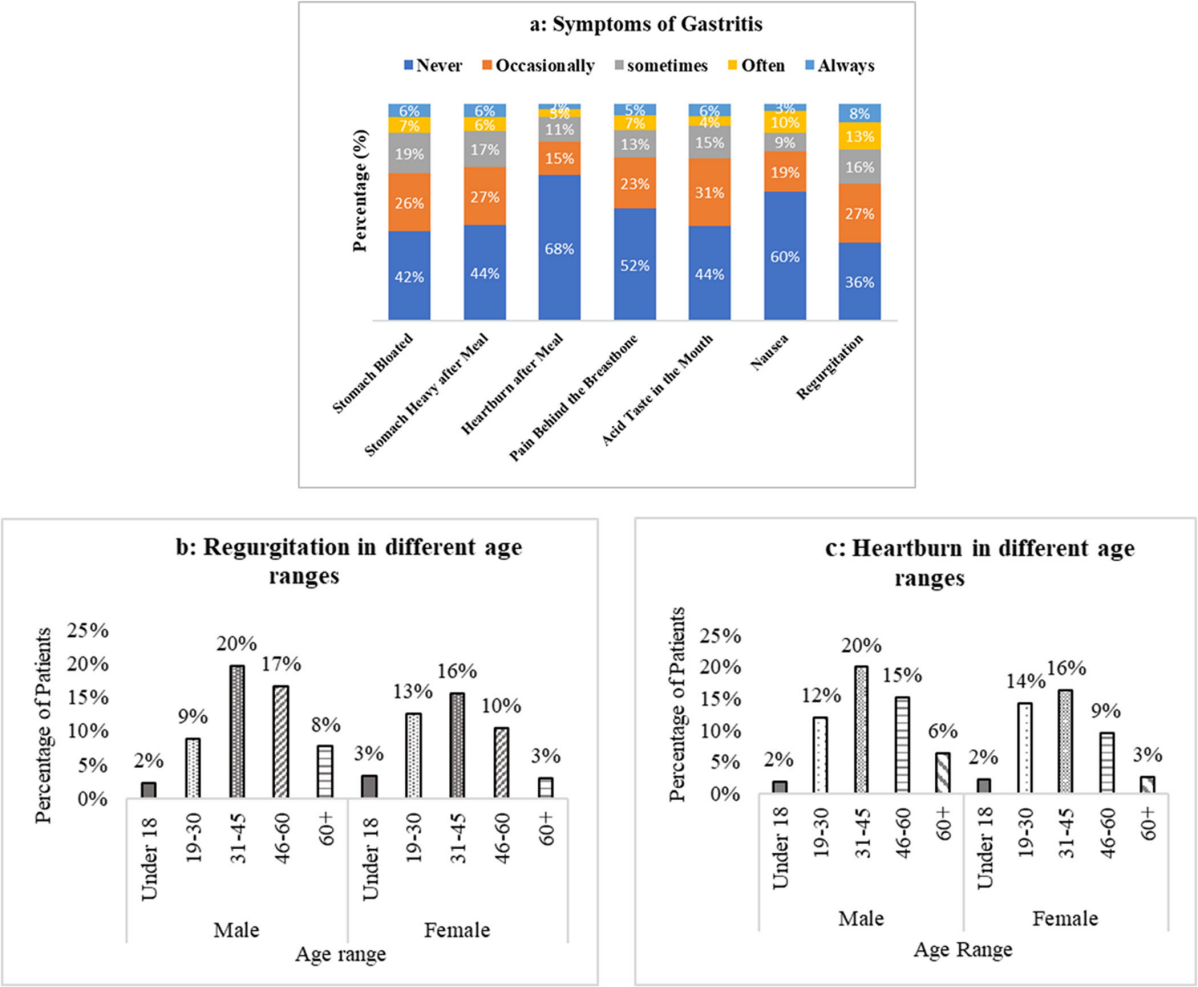
Common symptoms of gastritis reported by the participants (*n* = 1051). (a) Different symptoms of gastritis with five different frequencies (always, often, sometimes, occasionally, and never). (b) Prevalence (percentage of patients) of heartburn in different age ranges and sex. (c) Prevalence (percentage of patients) of regurgitation in different age ranges and sex.

Regurgitation (64%) is the most prevalent cause of common symptoms, followed by stomach bloating, heavy stomach, and an acidic taste in frequency of occurrence. On the other hand, heartburn ranks as the least prevalent cause in this study. (Figure 3a). Patients between the ages of 31 and 45 were the most likely (males 20% and females 16%) to suffer from regurgitation and heartburn (Figure 3b,c).

### 3.4 Self‐Medication

**Figure 4:**
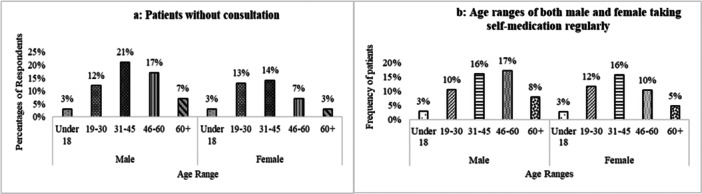
Self‐medication of participants. (a) Percentage of gastric patients taking the medication without prescription/consultation in different age ranges and sex. (b) Percentage of participants in different age ranges and sex who have taken self‐medication regularly.

Incidentally, 51% of participating patients took gastric medication without a prescription or consultation with healthcare providers. Among them, male patients were significantly (*p* < 0.001) more advanced in taking self‐medication between the ages of 31 and 60 than females (Figure 4a). Of the 1051 participants, 35% (n = 373) of patients took self‐medication regularly, 32%, 10%, and 5% took it occasionally, sometimes, and often, respectively. In the regular self‐medication group of patients, most of the males between the ages of 46 and 60 (17%) and females between 31 and 45 (16%) took self‐medication (Figure 4b).

### 3.5 Treatment

**Figure 5:**
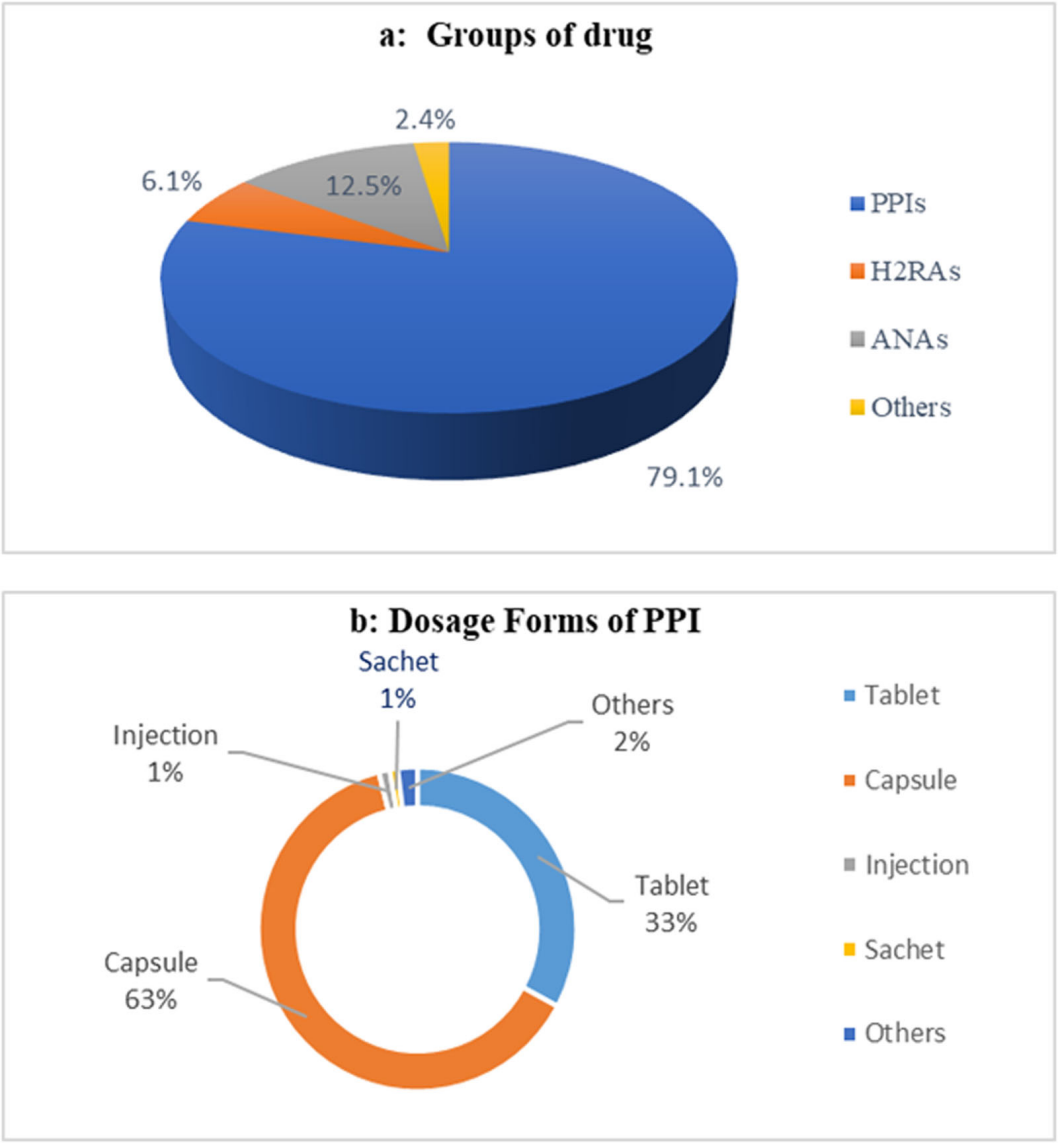
Treatment of patients. (a) Major groups of drugs used by the participants. (b) Common dosage forms of PPI drugs used by the participants.

In this survey, we observed four significant groups or types of drugs consumed by the patients. The majority of the people were taking PPIs (79.1%), followed by antacid preparations (ANAs, 12.5%), histamine‐2 blockers (H_2_RAs, 6.1%), and other traditional practices such as homeopathy, herbs, and promotility agents, which were less than 2.4% (Figure 5a).

For this significant group of PPIs, multiple dosage forms of gastric drugs are available in the Bangladeshi pharmaceutical market. Among them, oral dosage forms (capsules 63% and tablets 33%) are more common than others (injections, sachets, and suspensions) in Figure 5b.

### 3.6 Frequency of Taking Medicine

**Figure 6:**
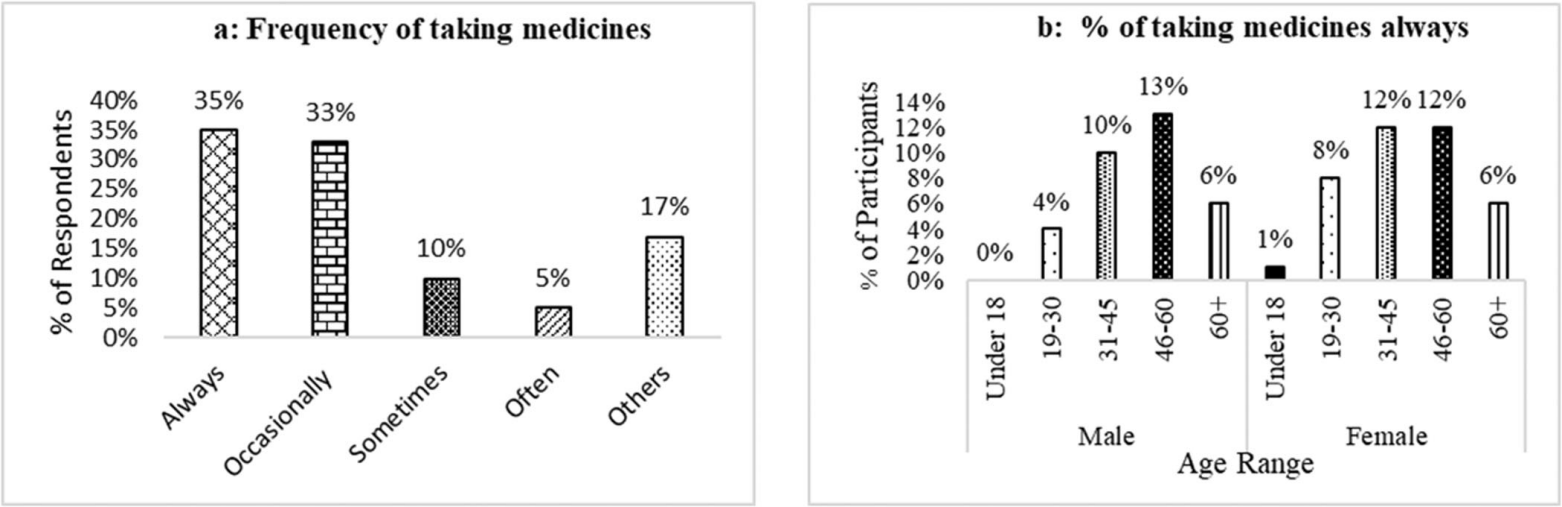
Frequency of taking medications. (a) Percentage of frequency of taking gastric medicine. (b) Percentage of taking medicine according to age ranges and sex, taking gastric medicines always (35% participants).

Eighty‐one percent of participants took gastric medication; the remaining 19% were with other medicines (Figure 6a). Among the always‐taking patients, male or female, people between 46 and 60 were the most frequently taking people, followed by the 31‐ to 45‐year‐old age group (Figure 6b).

## 4 Discussion

The worldwide prevalence of GI disease is about 40% in people at any given time; among these people, two‐thirds will have chronic and fluctuating symptoms [8, 12]. This study's symptomatic data (*N* = 1051) were taken using valid questionnaires for acute and chronic gastritis with different symptoms. In general, males are more prone to suffer from FGID than women, increasing with age [13]. This investigation revealed a similar pattern of results: males were significantly more suffering patients than females aged 31–45 (Figure 1). In developing countries, chronic gastritis is a relatively common disease. *H. pylori* infection is about 69% in Africa, 78% in South America, 51% in Asia, and 59.1%, particularly in Bangladesh [7, 13]. Sometimes, there are no clinical manifestations of acute gastritis, but the onset of pain behind the breast bone, nausea, and vomiting are reported to accompany gastritis. Many people have no symptoms or develop minimal symptoms of dyspepsia. If left untreated, the picture can progress to chronic gastritis. A history of smoking [13], alcohol consumption, NSAID or steroid use, allergies, radiation therapy, or gallbladder disorders should all be considered [7].

From the study, we also found some other causes, such as a sedentary lifestyle (45%), obesity (28%), stress (12%), smoking/drinking alcohol (8%), sedentary lifestyle, and taking gastric medication with other medicines (6.58%). An inactive lifestyle is unsuitable for overall health and digestion [14]. Scientific studies reported a correlation between obesity and gastritis, gastric ulcers, and increased risk of developing gallstones [15, 16, 17]. Similarly, stress and anxiety affect mental and digestive health, particularly gut microbiota, which correlates with the recent establishment theory of gut‐brain interaction [18]. Also, it decreases gastric renewal, leading to atrophy of the gastric mucosa, reduced blood flow to the stomach, and makes the stomach more prone to acid pepsin ulceration and hyperacid secretion [16]. Tobacco or alcohol may reduce pressure or prolong acid exposure duration and imply gastritis symptoms [15]. Taking gastric medicine with other medications, especially with NSAIDs, is higher in number, and if they don't take gastric treatment, they will suffer from gastritis [19]. Particularly in Bangladesh, there are also some potential influences for the prevalence of gastritis, such as culturally taking excessive chili, turmeric, and acidic foods, poor hygiene, malnutrition, vitamin deficiency, and certain genetic polymorphisms (e.g., IL‐1β and TNF‐α), which may make Bangladeshis more susceptible to gastritis. Results of this study revealed that a sedentary lifestyle and obesity are the most active causes following stress, smoking, and taking other medication, which supports those previous reports (Figure 2).

Symptoms are generated through a complex interplay between factors such as gut dysbiosis, changed mucosal immune response, altered gut signaling, and central nervous system dysregulation in gut signaling and regulation of motor function [18]. In this survey, some common gastritis symptoms can be seen in most participants with different frequency, such as regurgitation (64%), stomach bloating (58%), pain behind the breastbone (56%), nausea and vomiting (40%), and heartburn (32%) (Figure 3). The overlapping symptomatic data show that 64% and 58% of participants suffered from regurgitation and stomach bloating, respectively, because of gastritis, which is a strong indicator of GERD [20]. Lifestyle factors (stress, cigarette/smoking, specific exercise, and other medication use) also significantly affect heartburn in older age; thus, the severity of heartburn increases with age [10]. Participants between the ages of 31 and 60 suffered the most from regurgitation (males 36% and females 27%) and heartburn (males 36%, females 24%), whereas males suffered significantly more than females (Figure 3b,c). Also, the literature review shows patients with bloating, chest pain, nausea and vomiting, and acid taste due to reflux [8].

Patients take medicine with or without consultation from doctors. A consultation is a secret and intimate discussion between a doctor and a patient [21]. Consultation with pharmacists means the opportunity to educate patients about medicines and protect them from problems associated with new medications by discussing possible side effects, contraindications, and the importance of following the directions of a physician. Most consultations last 1–3 min, and a private consulting area is needed [22]. 49% of participants reported taking the medicine according to the doctor's advice. That means the consultation for gastric symptoms in the population is very low and again males from the age group 31–60 were taking medicines without consultation more than females (Figure 4a).

Most of the survey participants depended on self‐medication. By the definition of the International Pharmaceutical Federation (FIP) and the World Self‐Medication Industry (WSMI), self‐medication is the use of nonprescription medicines by patients' initiative [23]. According to the literature review, digestive problems, diarrhea, pain, and so on, are the common symptoms, and antacids, PPIs, NSAIDs, antidiarrheal drugs, and so on, are the common medications of self‐medication. Also, self‐medication is more common among people of all ages in developing countries than in developed countries [24]. This survey of OTC drugs found similar (51%) severity in Bangladesh, a developing country. In this survey, 31‐ to 60‐year‐old patients (males 33% and females 26%) took self‐medication regularly, unlike other age group patients (Figure 4b).

For treating most digestive disorders, mainly three modern groups of drugs, PPIs (79.1%), ANAs (12.5%), and H_2_Ras (6.1%) are used (Figure 5a). Commonly used PPIs are omeprazole, esomeprazole, lansoprazole, pantoprazole, and rabiprazole. Sometimes, PPIs can cause renal complications and community‐acquired pneumonia. Literature reviews reported a possible association between long‐term use of PPI and increased risk of cancer [25]. The most popular H_2_ blockers are cimetidine, ranitidine, famotidine, and nizatidine. Although H_2_ blockers have an excellent safety profile after prolonged use, they show many problems like gynecomastia, impotence, anemia, neutropenia, confusion, restlessness, and headache.

H_2_ blockers and PPIs are successfully used to treat PUD and GERD. In treating esophageal reflux disease, H_2_ blockers are inferior to PPIs because some acid is still produced when using H_2_ blockers, whereas antacids are ineffective. PPIs block the PP inside parietal cells and give more significant acid suppression than H_2_ blockers. PPIs have a superior effect on symptom resolution and mucosal healing over other drugs for prolonged periods. They are cost‐effective; most participants (79.1%) chose them as the first‐line drug in treating gastritis. So, PPIs are more effective and popular than others [26].

ANAs are inexpensive, readily available, and safe among gastric medications. It shows its action within 5 min after ingestion and lasts 30 min. Examples of some ANAs are antacids containing Mg (OH)_2_ + Al (OH)_3_ + simethicone active ingredients; Na_2_CO_3_, NaHCO_3,_ and CaCO_3_ comprising ENO. Despite having many advantages, it also causes milk‐alkali syndrome, renal insufficiency, diarrhea, hypermagnesemia, encephalotomy, and so on [27].

But some people prefer medicinal herbs, namely Black seeds [28], ginger [29], isabgol (psyllium) [30], anise, Shulamardan [31], and peppermint, from traditional medicine. A high dose of every medication has some side effects with its therapeutic effect [32]. So, although herbs can help reduce gas, stomach bloating, and ulcers and relieve stomach pain, patients should be careful about their use. Moreover, many homeopathy treatments are also being used as remedies for gastritis.

Several dosage forms of these drugs are available according to the patient's needs and based on different pharmacological properties. The solid dosage form is significantly popular for PPIs (Capsule 63%, tablet 33%, injection 1%, sachet 1%, and others 2%) group drugs. Still, various advantages of the oral drug delivery system are easy administration, shorter half‐life, unit dosage form, lightest and most compact, readily available, do not have stability problems, which makes it convenient for all [33]. The survey also shows that the oral route is friendly for approximately 96% of the participants (Figure 5b).

In this survey, we found various frequencies of taking gastric medicines, which is also considerable for maintaining good health. 35% and 33% of participants took gastric medicines always and occasionally, respectively (Figure 6). Taking medications without proper medical consultation or taking the wrong route can also lead to drug interactions. Self‐medication also increases the potential for drug abuse and addiction. So, a doctor's consultation is still the best solution, instead of taking medicine without consultation. Some steps should also be taken, such as making patients aware of the risk factors of OTC medication, because gastric drugs are considered OTC drugs [34].

While this survey provides valuable insights into the prevalence, causes, symptoms, and medications for gastric diseases and FGIDs in Bangladesh, there are some inherent limitations, such as its cross‐sectional design, reliance on self‐reported data, lack of follow‐up diagnostics, sampling limitations, limited control of confounding factors, potential measurement errors, and so on.

Despite these limitations, the survey still provides valuable preliminary data on gastric diseases and FGIDs in Bangladesh. The survey on gastritis in Bangladesh has substantial clinical consequences since it provides information about the prevalence, risk factors, and health‐seeking behaviors related to this prevalent GI illness. Identifying eating habits, illness rates (especially *H. pylori*), and environmental triggers can assist healthcare providers in better understanding area epidemiology. This information is critical for personalizing public health activities, enhancing diagnostic techniques, and developing treatment procedures.

Furthermore, such a study can help guide the taking of initiatives for raising awareness about prevention, timely management, and the potential long‐term consequences of untreated gastritis, such as peptic ulcers or stomach cancer. However, Future research should consider longitudinal studies, clinical diagnoses, and larger representative samples to strengthen the findings. We need a large‐scale, follow‐up study of gastric patients to explore clear insights.

## 5 Conclusion

The findings of this study contribute to a better understanding of the current causes, epidemiology, diagnosis, symptoms, and treatment of gastritis (drug therapy) in Bangladesh. Dyspeptic symptoms are widespread in gastritis patients and harm their health‐related quality of life. To control gastritis, we should follow some routines such as eating small meals slowly and on time; avoiding smoking, overusing painkillers, and eliminating some foods that cause gastritis problems; drinking tea with some herbal like anise, ginger, and pepper may aid digestion and reduce gastric pain; and doing exercise and breathing practices for stress management, which is a major cause of gastritis nowadays. Additionally, we should avoid the misuse of medicines and self‐medicating without proper guidance and follow the advice of competent health professionals.

## Author Contributions

**Md. Shoriful Islam:** conceptualization, writing – original draft, writing – review and editing, supervision, software, methodology, validation, funding acquisition, project administration, resources, visualization. **Rashni Agarwala:** data curation, investigation, validation, formal analysis, writing – review and editing, writing – original draft, methodology, software. **Sozoni Khatun:** investigation, writing – review and editing, validation, formal analysis, data curation. **Tonima Enam:** data curation, formal analysis, validation, investigation, writing – review and editing. **Ayesha Siddika:** investigation, validation, formal analysis, writing – review and editing. **Tasfia Saffat:** investigation, validation, data curation, writing – review and editing. **Mst. Israt Jahan:** investigation, validation, data curation, writing – review and editing. **Afia Ibnath Shimki:** investigation, data curation, writing – review and editing. **Azifa Akter:** data curation, investigation, writing – review and editing. **Mahajabin Snigdha:** investigation, data curation, writing – review and editing. **Rubaia Tasmin:** investigation, data curation, writing – review and editing.

## Conflicts of Interest

The authors declare no conflicts of interest.

## Transparency Statement

The lead author, Md. Shoriful Islam affirms that this manuscript is an honest, accurate, and transparent account of the study being reported; that no important aspects of the study have been omitted; and that any discrepancies from the study as planned (and, if relevant, registered) have been explained.

## Supporting information

Supplementary data.

## Data Availability

The data that support the findings of this study are available in Supporting Information S1 of this article.
