# Supplementary material for: A Cross‐Sectional Study on Gastric Diseases and FGIDs in Bangladesh: Insights Into Causes, Symptoms, and Medications
Source: Health Sci Rep. 2025 Aug 19;8(8):e71188. doi: 10.1002/hsr2.71188 (PMC12365003; doi:10.1002/hsr2.71188)
Supplement: Supplementary file 1 — Supplementary data. [file HSR2-8-e71188-s001.pdf]

Supplementary data:

A Cross-Sectional Study on Gastric Diseases and FGIDs in Bangladesh: Insights into Causes, Symptoms, and Medications

Authors:

*Md. Shoriful Islam[1], * Rashni Agarwala[1], Sozoni khatun[1], Tonima Enam[1], Ayesha Siddika[1], Tasfia Saffat[1], Mst. Israt Jahan[1], Afia Ibnath Shimki[1], Azifa Akter[1], Mahajabin Snigdha[1], Rubaia Tasmin[1]

Affiliations: Department of Pharmacy, Islamic University, Bangladesh

Corresponding author details: Md. Shoriful Islam, Assistant Professor, Department of Pharmacy, Islamic University, Bangladesh. Email: shoriful@pharmacy.iu.ac.bd

Supplementary attachment Table 1 (Table S1): List of Hospitals/Clinics from which we collected our data:

District	Hospital/ clinic name
Kushtia	Kushtia General Hospital, Ad-din Hospital Kushtia, Bheramara Health Complex
Jhenaidah	Sadar Hospital, Jhenaidah
Jessore	Jashore General Hospital
Rajshahi	Rajshahi General Hospital and Diagnostic Center, Rajshahi Royal Hospital Pvt. Ltd.
Bogura	Sherpur Upazila Health Complex
Rajbari	Rajbari General Hospital, Popular clinic
Dinajpur	Dinajpur General Hospital; Rogmukti Clinic, Golkuti, Dinajpur; Tista Clinic, Balubari, Dinajpur
Dhaka	Kurmitola General Hospital

Supplementary attachment Image 1 (Image S1): Application format for the hospitals/clinics

Date: / /2022

Director

……………………………………hospital/ clinic

Sub: Prayer for permission on a survey of gastritis diseases & drug therapy.

Sir, we are students of the Department of Pharmacy, Islamic University, Bangladesh. A survey on "The Prevalence of Gastric Diseases and FGIDs in Bangladesh: Causes, Symptoms, and Medications: A Cross-Sectional Case Study" is carried out by the Department of Pharmacy, Islamic University, Bangladesh. So, we want to collect data from gastric patients under your observation at a Hospital/Clinic.

We, therefore pray and hope that you will permit us to collect data for a proper investigation of gastric diseases for our survey.

Yours obediently,
Students, Department of Pharmacy
Islamic University, Bangladesh.

Referred by
Arghy Prosun Sarkar
Chairman & Assistant Professor
Department of Pharmacy
Islamic University, Bangladesh.

Supervised by
Md. Shoriful Islam
Assistant Professor
Department of Pharmacy
Islamic University, Bangladesh

Supplementary attachment Image 2 (Image S2): Questionnaire format

Supplementary attachment (S3): Questionnaire format

Islamic University
Department of Pharmacy
Kushtia- 7000, Bangladesh

(Questionnaire-Based Survey on Gastric Diseases, FGIDs & Drug Therapy in Bangladesh

Serial No: **Address:**

Name: **Age:** **Sex:** **Weight:**

Type of Patient: Gastric Disease: ☐ Yes, If yes - ☐ No **If no -**

Taking Drug: ☐ With Other medication ☐ non-OTC ☐ OTC

Pregnant: ☐ Yes ☐ No

Types of Drugs

Brand Name	Generic Name	Group

Dosage Form: Tablet Capsule Syrup Sachet Injection Others

Is Patient Taking GERD Drugs: ☐ Known ☐ Unknown

Questions	Frequency				
	Never	Occasionally	Sometimes	Often	Always
Do you get heartburn?					
Does your stomach get bloated?					
Does your stomach ever feel heavy after meals?					
Do you get heartburn after meals?					
Acid taste in the mouth?					
Burning behind the breastbone?					
Pain behind the breastbone?					
Is the patient taking an acidity drug?					
Tension/ Stress					
Smoking					
Alcohol					

Patient's History:

Purpose of this Survey-based research:

To study Gastric Diseases, Functional Gastrointestinal Disorders (FGID) disorders & Drug Therapy in Bangladesh that frequently occur in Bangladesh and to determine the effect of food and drug therapy

"I hereby declare that the above-mentioned information is correct and trustable."

Signature of Patient
Date:
Contact no:

Signature of Data Collector
Date:
Contact no:

Date: / /2022
